# Homocysteine causes neuronal leptin resistance and endoplasmic reticulum stress

**DOI:** 10.1371/journal.pone.0278965

**Published:** 2022-12-13

**Authors:** Arini Isnani Preninka, Karen Kuriya, Kyosuke Yazawa, Michiko Yoshii, Yuhki Yanase, Ralf Jockers, Julie Dam, Toru Hosoi, Koichiro Ozawa

**Affiliations:** 1 Department of Pharmacotherapy, Graduate School of Biomedical and Health Sciences, Hiroshima University, Hiroshima, Japan; 2 Faculty of Pharmaceutical Sciences, Department of Clinical Pharmacology, Sanyo-Onoda City University, Yamaguchi, Japan; 3 Université Paris Cité, CNRS, INSERM, Institut Cochin, Paris, France; Amity University, INDIA

## Abstract

Abnormally high serum homocysteine levels have been associated with several disorders, including obesity, cardiovascular diseases or neurological diseases. Leptin is an anti-obesity protein and its action is mainly mediated by the activation of its Ob-R receptor in neuronal cells. The inability of leptin to induce activation of its specific signaling pathways, especially under endoplasmic reticulum stress, leads to the leptin resistance observed in obesity. The present study examined the effect of homocysteine on leptin signaling in SH-SY5Y neuroblastoma cells expressing the leptin receptor Ob-Rb. Phosphorylation of the signal transducer and activator of transcription (STAT3) and leptin-induced STAT3 transcriptional activity were significantly inhibited by homocysteine treatment. These effects may be specific to homocysteine and to the leptin pathway, as other homocysteine-related compounds, namely methionine and cysteine, have weak effect on leptin-induced inhibition of STAT3 phosphorylation, and homocysteine has no impact on IL-6-induced activation of STAT3. The direct effect of homocysteine on leptin-induced Ob-R activation, analyzed by Ob-R BRET biosensor to monitor Ob-R oligomerization and conformational change, suggested that homocysteine treatment does not affect early events of leptin-induced Ob-R activation. Instead, we found that, unlike methionine or cysteine, homocysteine increases the expression of the endoplasmic reticulum (ER) stress response gene, a homocysteine-sensitive ER resident protein. These results suggest that homocysteine may induce neuronal resistance to leptin by suppressing STAT3 phosphorylation downstream of the leptin receptor via ER stress.

## Introduction

Obesity has become a global concern because of its association with metabolic diseases such as diabetes, hypertension, and dyslipidemia. Leptin plays an important role in the regulation of food consumption and hunger and controls energy metabolism. Leptin signaling is triggered by the binding of leptin to the Ob-Rb receptor, followed by the activation of Janus Kinase 2 (JAK2), which leads to the activation of the Signal Transducer and Activator of Transcription 3 (STAT3), known as the JAK2-STAT3 signaling pathway [1,2]. Insensitivity to the action of leptin, termed “leptin resistance”, is a potential mechanism underlying the development of obesity [3,4]. The mechanism of leptin resistance remains unclear, but the suppressor of cytokine signaling 3 (SOCS3) and the tyrosine phosphatase 1B, have been reported to block leptin induce-signal transduction [5,6] and are known negative regulators of ER stress [7]. Alteration of the JAK2-STAT3 pathway and enhancement of SOCS3 expression are crucial factors in leptin resistance in the central nervous system [8].

The endoplasmic reticulum (ER) is an organelle where protein folding and synthesis occur [9]. When the ER cannot handle an overwhelming amount of protein, which must be moved into the Golgi bodies, homeostasis in the ER lumen is disrupted, and this condition is called “ER stress” [10]. We and other groups have reported that ER stress may play a role in inducing leptin resistance [3,11], and could affect the result in the development of obesity [12,13]. Saturated fatty acid-palmitate has been suggested to increase ER stress by binding to the Toll-like receptor (TLR-4), and thereby trigger leptin resistance contributing to the development of obesity [14,15]. ER stress initiates unfolded protein response (UPR) signaling [16] by inducing a molecular chaperone called glucose-regulated protein 78 [17]. Accumulation of unfolded proteins in the ER activates stress sensor proteins such as inositol-requiring kinase 1α, double-stranded RNA-activated protein kinase R-like ER kinase, and activating transcription factor 6 (ATF6). These ER stress sensor proteins activate UPR-related genes via X-box binding protein 1 or decrease the translation by activating ATF4 [18]. Although cells have the capacity to adapt to ER stress, they encounter apoptosis when their capacity is overloaded [19].

The homocysteine-responsive ER-resident protein (HERP), located in the ER membrane, plays an important role in ER-associated protein degradation (ERAD) [20]. It is also considered a marker of ER stress-related apoptosis as it is linked to the ERAD pathway that functions on ubiquitin-and proteasome-dependent degradation to discard the unfolded proteins [21]. HERP, which has a ubiquitin-like domain, has also been used to examine protein folding in the ER membrane since Herp has been suggested to ameliorate the folding of ER proteins and to dampen ER protein load [22]. When ER stress occurs, HERP is highly induced [23].

Homocysteine is formed by methionine metabolism [24] through the chronological synthesis of S-adenosylmethionine and S-adenosylhomocysteine. Several reports have suggested the involvement of homocysteine and its metabolites in obesity. Changes in plasma betaine, choline, and glycine in the development of obesity may result in changes in the homocysteine/methionine metabolism [25–28]. Plasma homocysteine showed significant accumulation in obesity [29,30]. Homocysteine has been reported to participate in DNA methylation in the methionine cycle, which is involved in epigenetic modification of the genome [31,32].

Our previous findings and those of others have found ER stress to be one of the factors inducing leptin resistance. In addition, we previously reported that homocysteine may induce leptin resistance using HEK293 cells expressed leptin receptors [3]. However, the mechanism of homocysteine-induced leptin resistance remains unclear. Furthermore, it is unknown whether homocysteine can cause leptin resistance in neuronal cells.

## Materials and methods

### Reagents

Homocysteine (Hcy) was obtained from SIGMA (St. Louis, MO), methionine was obtained from Sigma-Aldrich Cheme, cysteine was obtained from Wako Pure Chemical Industries, Ltd, anti-phospho STAT3 was obtained from Cell Signaling Technology, anti-STAT3 was obtained from Santa Cruz Biotechnology, anti-HERP was obtained from Proteintech.

### Cell lines

#### SHSY5Y cell line

Human neuroblastoma cell lines: SH-SY5Y neuroblastoma cell line, which stably expressed Ob-Rb leptin receptor (SH-SY5Y Ob-Rb cells), was established previously [33]. SH-SY5Y Ob-Rb cells were cultured in Dulbecco’s modified Eagle’s medium supplemented with 10% (v/v) heat-inactivated fetal calf serum at 37°C in humidified 5% CO_2_ and 95% air.

#### HEK293T cell line

Human embryonic kidney 293T cells were cultured in Dulbecco’s modified Eagle’s medium supplemented with 10% (v/v) heat-inactivated fetal calf serum at 37°C in humidified 5% CO_2_ and 95% air.

### Cell culture

Homocysteine was dissolved directly into the medium. The cells were stimulated with homocysteine 10 mM for 4 hours by replacing the medium containing homocysteine.

### Western blotting

Cells were washed with ice-cold phosphate buffer saline and then lysed with lysis buffer containing 10 mM HEPES-NaOH (pH 7.5), 150 mM NaCl, 1 mM ethylenediaminetetraacetic acid, 1 mM Na_3_VO_4_, 10 mM NaF, 1% NP-40, 10 μg/mL aprotinin, 10 μg/mL leupeptin, and 1 mM phenylmethylsulphonyl fluoride. Following this, the lysates were centrifuged at 15,000 rpm at 4°C for 20 minutes. After centrifugation, the supernatants were collected. The samples were boiled with Laemmli buffer for 3 minutes, fractioned using SDS-PAGE, and transferred to nitrocellulose membranes at 4°C. These membranes were incubated with anti-phospho STAT3 (Tyr705; dilution 1:2000; Cell Signaling Technology), anti-STAT3 (dilution 1:1000; Santa Cruz Biotechnology), anti-glyceraldehyde 3-phosphate dehydrogenase (dilution 1:1000; Proteintech), and anti-HERP (dilution 1:1000; Proteintech) antibodies, followed by an anti-horseradish peroxidase-linked antibody. Finally, peroxidase binding was detected using a chemiluminescence reagent.

### STAT3 reporter gene assay

SH-SY5Y Ob-Rb cells, transfected with STAT3 promoter-NanoLuc plasmid (Promega), were cultured at 96 well plate for 48 h. Cells were then treated with homocysteine (1–10 mM) and leptin (0.01μg/mL, equal to 0.6 nM) for 4 h, and luminescence signal was measured using Nano-Glo® Luciferase Assay System (Promega).

### Bioluminescence resonance energy transfer (BRET) analysis

The BRET assay was performed based on the literature (34). HEK293T cells were transfected with luciferase-fused leptin receptor (OBR-2K Rluc, energy donor) and with leptin receptor fused to YFP (OBR-2K YFP, energy acceptor) using polyethyleneimine (PEI) in a 12-well plate. Twenty-four hours after the transfection, cells were plated to a 96-well plate (PerkinElmer, Culture Plate^TM^-96) and grown for 20 h. The cells were then pre-treated with homocysteine for 4 h and then with leptin (10 nM) for 30 min. For the BRET assay, the plate was washed with PBS, and Coelenterazine h (FUJIFILM Wako Pure Chemical cop., Japan) was added at final concentration of 20 μM. Luminescence signals were detected with filter settings for Donor: 495SP and Acceptor: 530LP at GloMax (Promega). The results were expressed as milliBRET units (mBRET unit) = {(YFP/Rluc)–(YFP/Rluc of cells expressing the donor alone)} × 1,000.

### Statistical analysis

**Fig 1:**
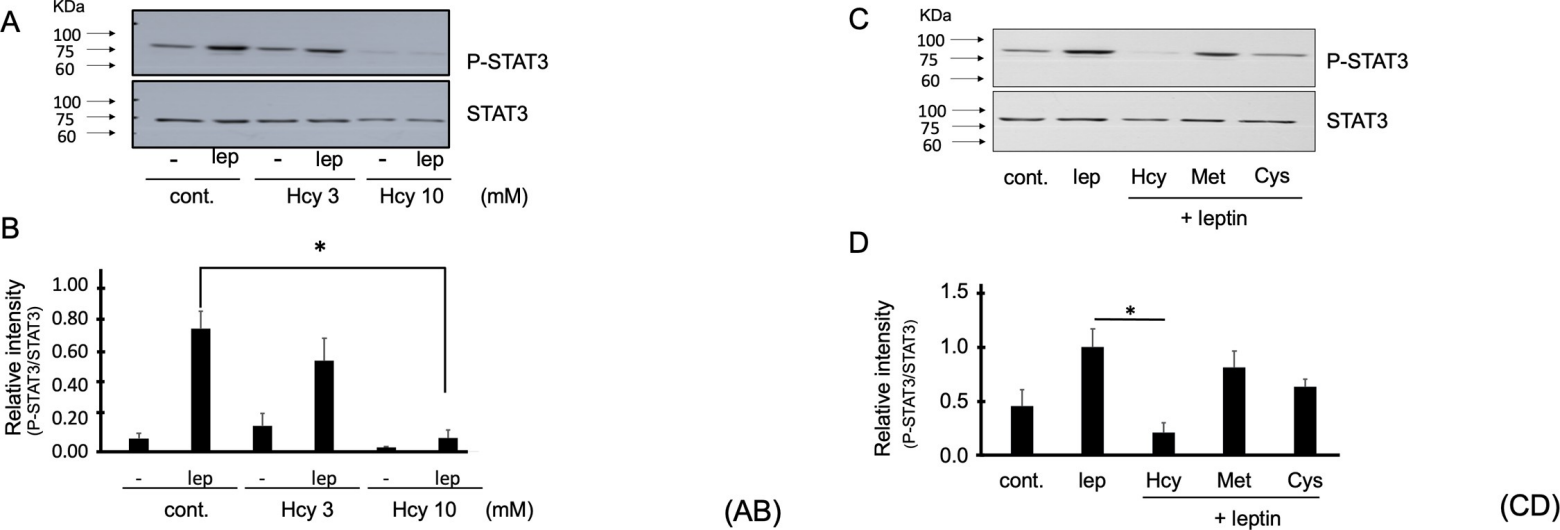
Homocysteine leads to significant inhibition of leptin receptor-induced signaling pathway. **(A-B) Homocysteine inhibited leptin-induced STAT3 phosphorylation.** SHSY5Y Ob-Rb cells were pretreated with homocysteine (Hcy; 3 and 10 mM) for 4 h and then stimulated with leptin (Lep; 0.01 μg/mL) for 15 min and leptin-induced STAT3 phosphorylation was analyzed by immunoblot. Statistical analysis used Dunnett’s post-hoc test following one-way ANOVA; *p< 0.05, n = 3–5. **(C-D) Methionine and cysteine were slightly inhibited leptin-induced STAT3 phosphorylation.** SHSY5Y Ob-Rb cells were treated with homocysteine (Hcy, 10 mM), methionine (Met, 10 mM) and cysteine (Cys, 10 mM) for 4 h and then stimulated with leptin (Lep; 0.01 μg/mL, 15 min), and leptin-induced STAT3 phosphorylation was analyzed. Statistical analysis used Turkey’s post-hoc test following one-way ANOVA; *p < 0.05, n = 3–5.

Results were expressed as the mean ± standard error of the stated value. Statistical analysis was performed using one-way analysis of variance followed by Dunnett’s test or Turkey’s test (Fig 1B).

## Results

### Homocysteine leads to significant inhibition of leptin receptor-induced signaling pathway

To assess the effect of homocysteine on leptin signaling, we pre-treated the SH-SY5Y-Ob-Rb human neuroblastoma cell line with homocysteine at 3 and 10 mM and analyzed the phosphorylation of STAT3 induced by leptin (0.01 μg/mL, equal to 0.6 nM). As expected, leptin treatment caused an increase in STAT3 phosphorylation in these neuronal cells. Pretreatment with 3 mM homocysteine slightly but not significantly inhibited leptin-induced STAT3 phosphorylation (p-value = 0.44), while 10 mM homocysteine significantly inhibited leptin phosphorylation (*p< 0.05) (Fig 1A and 1B).

To account for the chemical specificity of these effects, we also evaluated the impact of other compounds related to homocysteine, i.e., methionine (10 mM) and cysteine (10 mM), which have a structural formula similar to homocysteine. Whereas methionine did not inhibit and cysteine tended slightly to decrease leptin-induced phosphorylation of STAT3 homocysteine has a drastic inhibitory impact (*p<0.05) (Fig 1C and 1D). These results therefore, suggest that homocysteine specifically inhibits the leptin-induced STAT3 signaling compared to methionine and cysteine in a neuronal context.

### Homocysteine-mediated inhibition of leptin-induced STAT3 transcriptional activity assessed by STAT3 reporter gene assay

**Fig 2:**
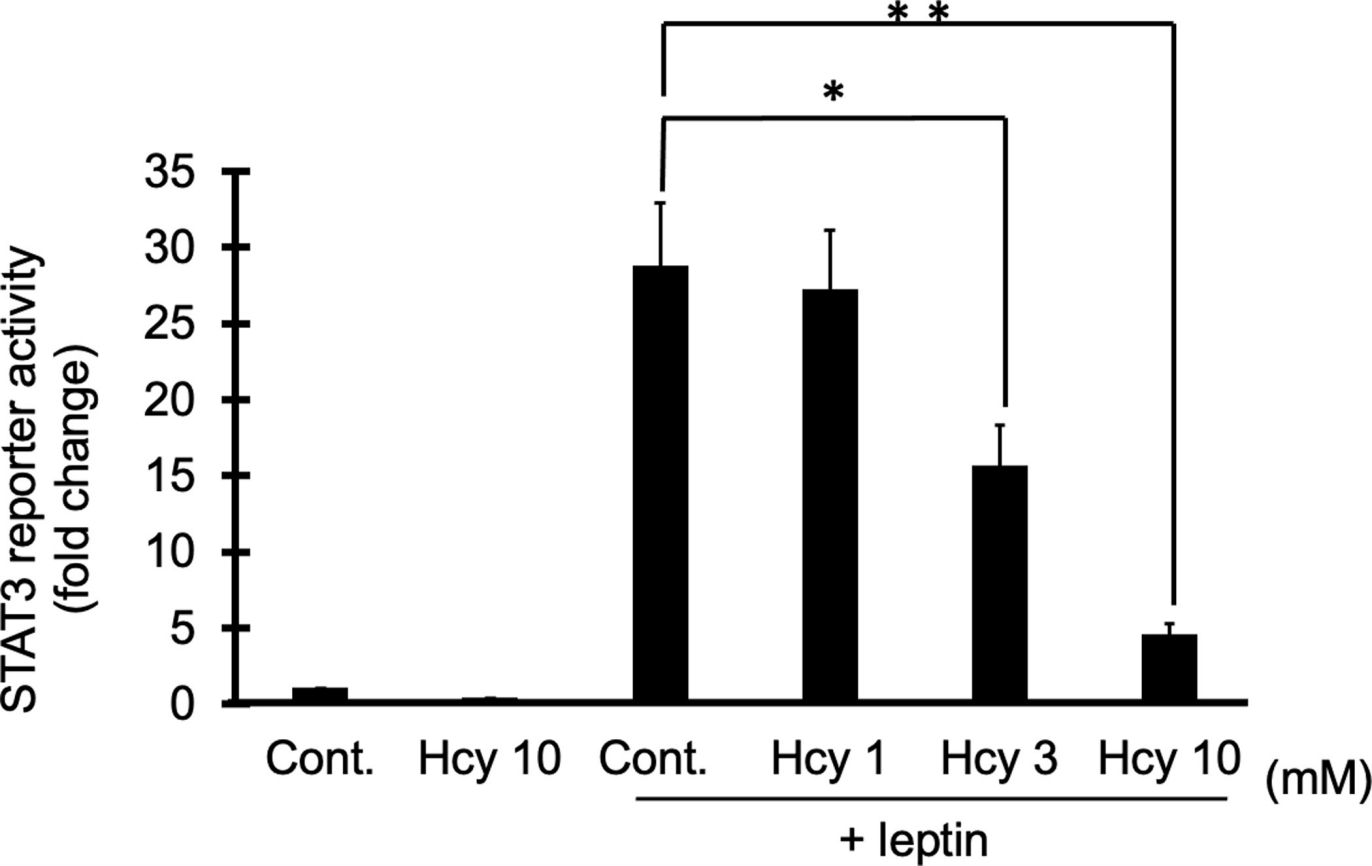
Homocysteine-mediated inhibition of leptin-induced STAT3 transcriptional activity assessed by STAT3 reporter gene assay. SH-SY5Y-Ob-Rb cells transfected with the STAT3-promoter-Nluc construct were treated with homocysteine (Hcy; 1–10 mM) and leptin (Lep; 0.01 μg/mL), and the luminescence signal is measured 4 hours after treatments. Data were expressed as relative luciferase activity, normalized with the control condition. *p < 0.05, **p < 0.01, n = 4.

We next evaluated the effect of homocysteine on leptin-induced STAT3 transcriptional activity with a STAT3 reporter gene assay in SH-SY5Y Ob-Rb cells. We used SH-SY5Y Ob-Rb cells stably expressing the STAT3 reporter gene and analyzed the effect of homocysteine on leptin-induced activation of STAT3 reporter. We found that leptin-induced STAT3 transcriptional activity was significantly inhibited by homocysteine in a dose-dependent manner (1–10 mM) (Fig 2). The results suggest that homocysteine also inhibits STAT3 transcriptional activity induced by leptin treatment.

### No impact of homocysteine on leptin-induced receptor conformational change using the leptin receptor BRET biosensor

To understand the mechanisms involved in homocysteine-induced leptin resistance, we evaluated the effect of homocysteine at the early events of the STAT3 signaling pathway, i.e. at the receptor activation stage, using a BRET biosensor. Upon stimulation by leptin, a protomer of the leptin receptor Ob-R fused to the energy donor, luciferase, and another protomer to the energy acceptor, yellow fluorescent protein (YFP), oligomerize and undergo a conformational change, causing an increase in the BRET signal, which is associated with the Ob-R activation state [34].

**Fig 3:**
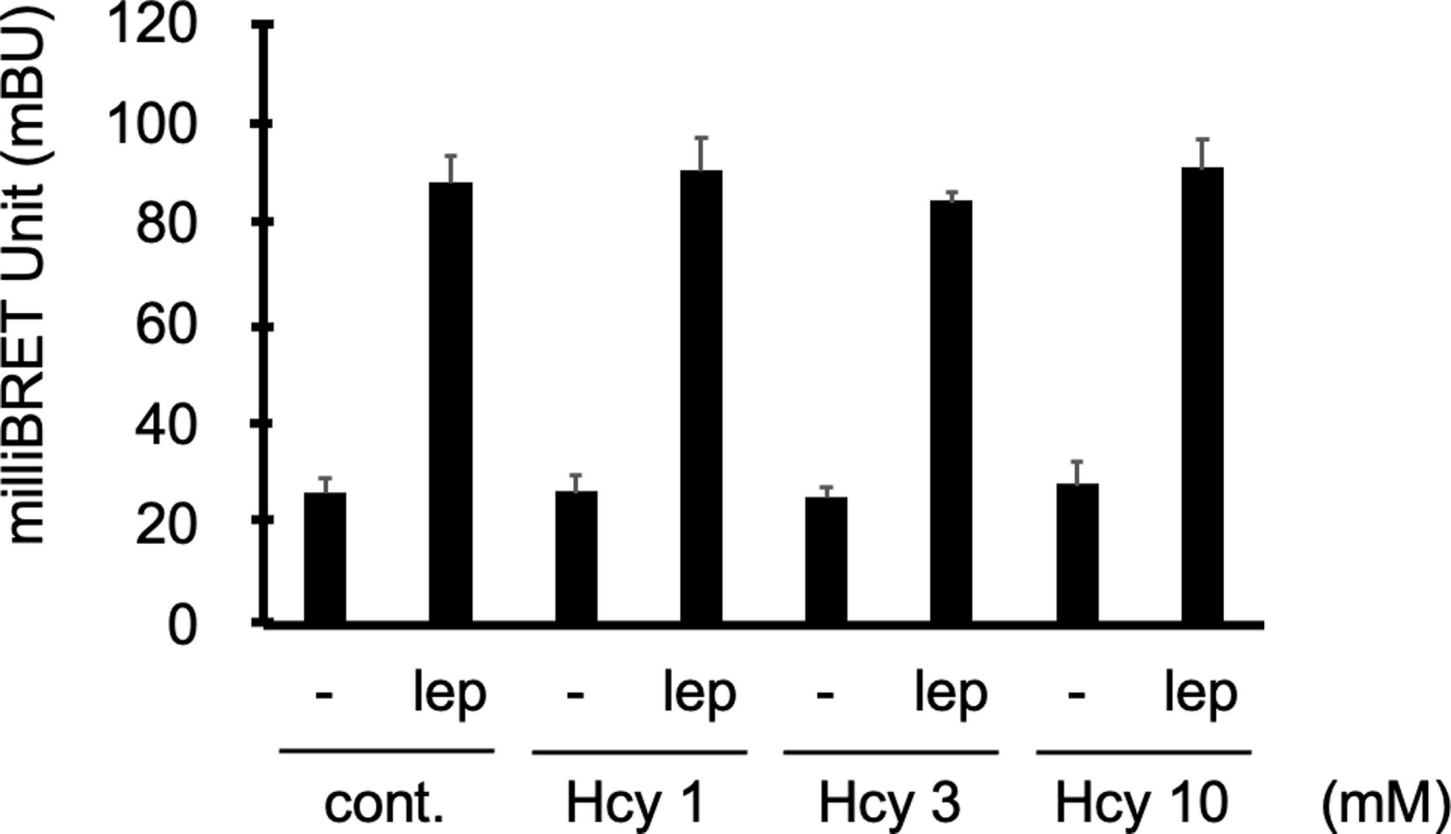
No impact of homocysteine on leptin-induced receptor conformational change using the leptin receptor BRET biosensor. HEK293T cells were transfected with leptin receptor fused to luciferase (OBR-2K-Rluc, energy donor) and leptin receptor fused to the yellow fluorescent protein (OBR 2K YFP, energy acceptor). The cells were then pre-treated with homocysteine (1–10 mM) and leptin (10 nM)-induced OBR conformational changes were detected by BRET assay n = 4.

Using stably transfected HEK293 cells with Ob-Rb receptor expression, we have previously shown that homocysteine can inhibit leptin-induced STAT3 phosphorylation in HEK293 [3]. We therefore, investigated whether homocysteine affected the leptin-induced activation signature of the Ob-R receptor using the Ob-R-BRET biosensor probe in HEK293T cells transfected with the donor (OBR-2K Rluc) and acceptor (OBR-2K YFP) constructs. We observed a 3-fold increase in BRET signal upon leptin stimulation, evoking leptin receptor oligomerization and conformational change. In contrast, homocysteine treatments (1–10 mM) did not modify this profile (Fig 3), suggesting that homocysteine probably does not affect the leptin-induced conformational change and oligomerization of the leptin receptor.

### Homocysteine induced endoplasmic reticulum stress

Next, we evaluated the mechanism of homocysteine-induced leptin resistance. One possible explanation is that homocysteine induces leptin resistance through the activation of ER stress. We found that homocysteine increased the expression level of the ER stress response gene, HERP at 4 and 8 hours, by 4-fold in SHY5Y neuronal cells. We analyzed whether homocysteine-induced HERP is known to be involved in ER stress.

**Fig 4:**
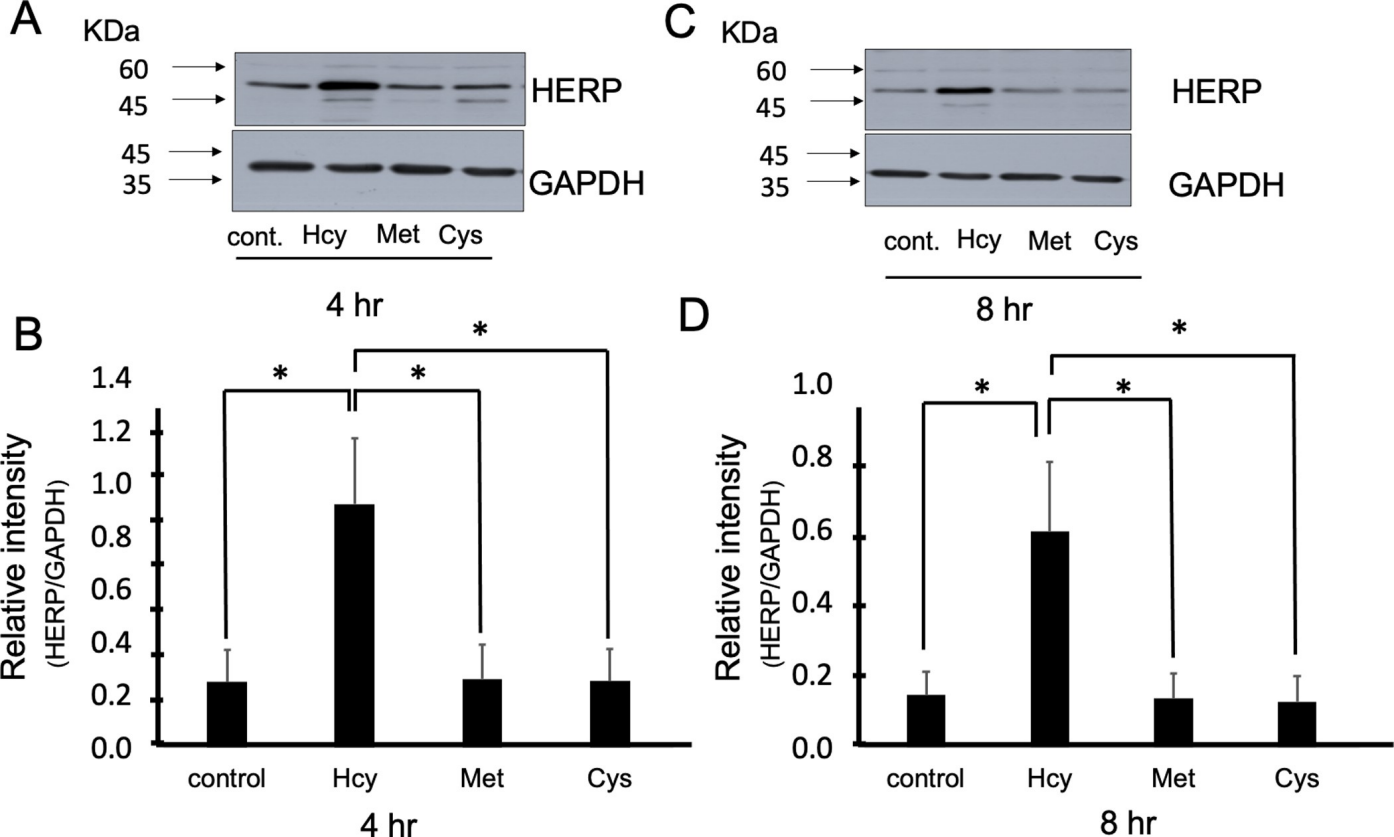
Homocysteine-induced endoplasmic reticulum stress. **(A-D)** SHY5Y-Ob-R cells were treated with homocysteine (Hcy), methionine (Met), and cysteine (Cys) for 4 and 8 hours at 10 mM, and the expression level of ER stress response gene, HERP, was analyzed by Dunnett test. *p < 0.05, n = 5.

In contrast, we did not detect an increase in HERP expression after treatment with cysteine and methionine (*p < 0.05, n = 5) (Fig 4A–4D). Thus, we suggest that homocysteine may participate in leptin resistance by inducing ER stress.

### Homocysteine has no impact on the IL-6 signaling pathway

**Fig 5:**
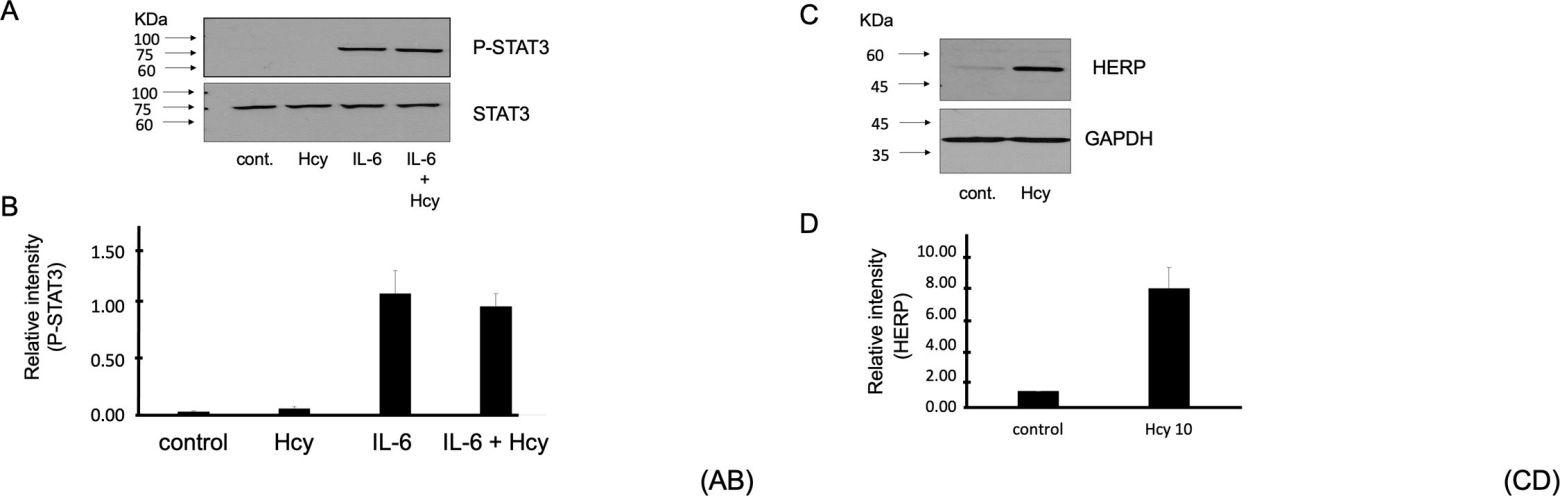
Homocysteine has no impact on the IL-6 signaling pathway. **(A-B)** HEK293T cells were treated with homocysteine (Hcy) for 4 hours at 10 mM, then stimulated with IL-6 (100 ng/mL) for 15 minutes. The level of phospho-STAT3 was analyzed. n = 5. **(C-D)** HEK293T cells were treated with homocysteine (Hcy) for 4 hours at 10 mM, then stimulated with IL-6 (100 ng/mL) for 15 minutes. The level of HERP was analyzed. n = 5.

To assess whether homocysteine has a specific impact on leptin signaling, we examined the effect of homocysteine on the IL-6-induced STAT3 pathway. We treated cells with homocysteine (10mM, 4 h) and analyzed interleukin-6 (IL-6) (100ng/mL)-induced STAT3 phosphorylation in the HEK293T cell line. We found that homocysteine did not affect IL-6-induced STAT3 signaling (Fig 5A and 5B). On the other hand, homocysteine can inhibit leptin-induced STAT3 phosphorylation in HEK293 cells stably transfected with Ob-Rb. Interestingly, the expression level of HERP as an ER stress response gene at 4 hours was significantly increased by homocysteine in HEK293T cells (Fig 5C and 5D), suggesting that homocysteine also likely contributes to ER stress in the HEK293T cell line, similar to the neuronal cells. Therefore, the impact of homocysteine on leptin-induced STAT3 phosphorylation appears to be specific to leptin receptor signaling since homocysteine, by inducing HERP expression, did not affect STAT3 phosphorylation mediated by IL6.

## Discussion

Leptin is a hormone that plays a crucial role in managing energy expenditure and food consumption by sending signals to the hypothalamus. Leptin binds to the Ob-Rb receptor, which then activates JAK2/STAT3 signaling pathway in neurons. Leptin resistance, in which the activation of its receptors at the central level no longer occurs correctly, is observed in obesity. Several possible mechanisms have been proposed to contribute to leptin resistance, including impaired leptin transport into the brain [35–38], a decreased availability of the Ob-R receptor on the cell surface [39–46], or induction of ER stress [3,11].

Homocysteine is a sulfur-containing, non-protein amino acid that naturally exists in the bloodstream. Its concentration is preserved by the re-methylation and transsulfuration pathways. The re-methylation pathway involves the conversion of homocysteine to methionine, whereas the transsulfuration pathway involves the conversion of homocysteine to cystathionine to form cysteine.

In this study, we discovered a functional link between homocysteine and leptin resistance in neuronal cells that were previously unknown. Homocysteine inhibits leptin signaling (inhibition of leptin-induced STAT3 phosphorylation and subsequent STAT3 transcriptional activity), suggesting that homocysteine may be involved in the leptin resistance phenomenon (Fig 1). We demonstrated that homocysteine reduces STAT3 signaling without affecting the leptin-induced activation state of Ob-R (oligomerization and conformational change). The mechanism by which homocysteine inhibits the leptin-induced STAT3 signaling pathway may involve the ER stress response gene, HERP, whose expression is increased upon homocysteine treatment. Thus, homocysteine may induce ER stress leading to leptin resistance in neuronal cells (Fig 4).

Other homocysteine-related compounds are methionine and cysteine, which have similar structural formulas. However, the results showed that only homocysteine strongly inhibited leptin signaling, whereas cysteine and methionine had no significant or very weak effect. Homocysteine and cysteine have thiols in their structural formula, whereas methionine has a sulfide. Therefore, it is possible that the thiol, not the sulfide, is involved in the development of leptin resistance.

Normal blood homocysteine levels range from 5 to 12 μM, and hyperhomocysteinemia is classified as mild (12–30 μM), moderate (30–100 μM), and severe (>100 μM) [47,48]. While in the in vitro experiment, the used concentrations vary. They are 0,1 mM, 1 mM, 2 mM, 3 mM [49,50], 2.5 mM [51], 5 mM [52], and 10 mM [53]. Also, we cannot exclude that local homocysteine concentrations around neurons may reach even higher values. High levels of homocysteine have been found in obese humans [54,55] and in obese mice [56]. A study also revealed that an increase in total body fat percent and lower lean mass are associated with increased homocysteine concentrations [57]. Because we would like to examine the leptin signal, we majorly focused on in vitro experiment, using SHSY5Y cell lines expressing Ob-Rb. In *in vivo* experiment, the Wistar of the National Institute of Nutrition obese (WNIN/Ob) seemed to be an appropriate animal model for obesity and other metabolic diseases. These rats are reported to show the characteristics of obesity such as insulin resistance and leptin resistance. They also are linked to decreased antioxidants which are then associated with aging problems [58,59]. It would be an important future subject to analyze *in vivo* effects using these models.

**Fig 6:**
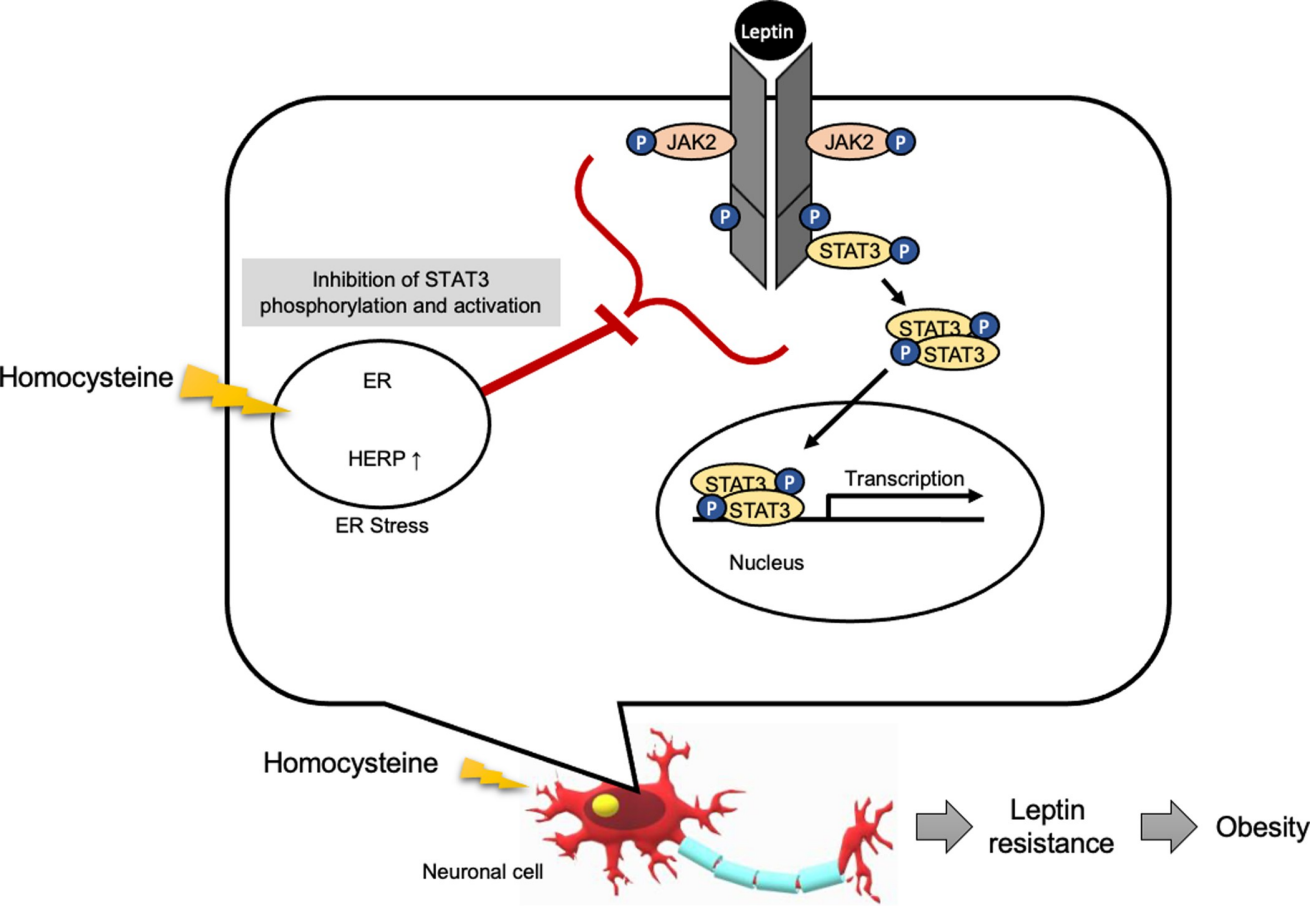
Model of homocysteine-induced leptin resistance. High concentrations of homocysteine trigger ER stress and inhibit the phosphorylation and transcriptional activity of STAT3 regulated by the anti-obesity hormone leptin. This mechanism may contribute to homocysteine-induced leptin resistance in neuronal cells.

In light of our results and the link between homocysteine and obesity, it is possible that homocysteine plays a key role in leptin resistance by inducing ER stress, contributing to the development of obesity and metabolic syndrome in conditions of hyperhomocysteinemia. In light of our results, we suggest that the induction of ER stress by homocysteine could constitute one of the mechanisms of leptin resistance (Fig 6).

A high level of homocysteine would trigger stress in the ER, which could subsequently alter specifically the JAK2-STAT3 signaling pathway regulated by the hormone leptin. When ER stress occurs, the inhibition of STAT3 phosphorylation increases [60–63]. We had shown that endoplasmic reticulum stress causes leptin resistance by decreasing the phosphorylation of STAT3. ER stress activates three signaling networks, i.e.; IRE1, PERK, and ATF6 [64]. PERK was reported to stimulate JAK-STAT signaling [65–67]. The IRE1 signaling pathway was also reported to be affected by the JAK-STAT signaling [68,69]. Even the mechanisms are still unknown, but the alteration of XBP1 was reported to disrupt the phosphorylation of STAT3. These reports suggest that IRE1/XBP1 and STAT3 signaling pathways may be closely related [70].

There was no report of homocysteine on leptin resistance in neuronal cells. We found for the first time that homocysteine causes leptin resistance in neuronal cells. We also found that the effect of homocysteine on leptin signaling may be specific to leptin receptor signaling, as we did not observe the inhibition of STAT3 signaling against IL-6. Targeting the homocysteine metabolic pathway and/or inhibiting the excessive action of homocysteine could have therapeutic value.

## Conclusion

Homocysteine may ameliorate the leptin signaling pathway by inhibiting JAK-STAT signaling that is regulated by the obesity hormone, leptin. In conclusion, homocysteine could cause neuronal leptin resistance by triggering endoplasmic reticulum stress, which would be one of the mechanisms of obesity.

## Supporting information

S1 Raw images (PDF)

## References

[1] Thon M, Hosoi T, Yoshii M, Ozawa K. Leptin induced GRP78 expression through the PI3K-mTOR pathway in neuronal cells. Sci Rep 4. 2014; 7096. doi: 10.1038/srep07096 25403445 PMC4235288

[2] Ghilardi N, Ziegler S, Wiestner A, Stoffel R, Heim MH, Skoda RC. Defective STAT signaling by the leptin receptor in diabetic mice. Proc Natl Acad Sci USA. 1996; 93(13): 6231–5. doi: 10.1073/pnas.93.13.6231 8692797 PMC39004

[3] Hosoi T, Sasaki M, Miyahara T, Hashimoto C, Matsuo S, Yoshii M, et al. Endoplasmic reticulum stress induces leptin resistance. Mol Pharmacol 74. 2008; 1610–1619. doi: 10.1124/mol.108.050070 18755873

[4] Münzberg H, Myers MG Jr. Molecular and anatomical determinants of central leptin resistance. Nat Neurosci. 2005; 8(5):566–70. doi: 10.1038/nn1454 15856064

[5] Bjørbaek C, Elmquist JK, Frantz JD, Shoelson SE, Flier JS. Identification of SOCS-3 as a potential mediator of central leptin resistance. Mol Cell. 1998; (4): 619–25. doi: 10.1016/s1097-2765(00)80062-3 9660946

[6] Kaszubska W, Falls HD, Schaefer VG, Haasch D, Frost L, Hessler P, et al. Protein tyrosine phosphatase 1B negatively regulates leptin signaling in a hypothalamic cell line. Mol Cell Endocrinol. 2002; 195: 09–18. 10.1016/s0303-7207(02)00178-8. 12354677

[7] Popov D. Endoplasmic reticulum stress and the on site function of resident PTP1B. Biochem Biophys Res Commun. 2012; (422): 535–538. doi: 10.1016/j.bbrc.2012.05.048 22609202

[8] Son M, Oh S, Choi J, Jang JT, Choi CH, Park KY, et al. Attenuation of inflammation and leptin resistance by Pyrogallol-Phloroglucinol-6,6-Bieckol on in the brain of obese animal models. Nutrients. 2019; (11): 2773. doi: 10.3390/nu11112773 31739649 PMC6893810

[9] Oakes SA, Papa FR. The role of endoplasmic reticulum stress in human pathology. Annu Rev Pathol. 2015; (10): 173–94. doi: 10.1146/annurev-pathol-012513-104649 25387057 PMC5568783

[10] Wang M, Kaufman RJ. Protein misfolding in the endoplasmic reticulum as a conduit to human disease. Nature. 2016; 529 (7586): 326–35. doi: 10.1038/nature17041 26791723

[11] Ozcan L, Ergin AS, Lu A, Chung J, Sarkar S, Nie D, et al. Endoplasmic reticulum stress plays a central role in development of leptin resistance. Cell Metab. 2009; (9): 35–51. 10.1016/j.cmet.2008.12.004. 19117545

[12] Gruzdeva O, Borodkina D, Uchasova E, Dyleva Y, Barbarash O. Leptin resistance: underlying mechanisms and diagnosis. Diabetes Metab Syndr Obe. 2019; (12): 191–198. doi: 10.2147/DMSO.S182406 30774404 PMC6354688

[13] Schneeberger M, Dietrich MO, Sebastián D, Imbernón M, Castaño C, Garcia A, et al. Mitofusin 2 in POMC neurons connects ER stress with leptin resistance and energy imbalance. Cell. 2013; (155): 172–187. doi: 10.1016/j.cell.2013.09.003 24074867 PMC3839088

[14] Oh S, Son M, Choi J, Choi CH, Park KY, Son KH, et al. Phlorotannins from Ecklonia cava attenuates palmitate-induced endoplasmic reticulum stress and leptin resistance in hypothalamic neurons. Mar Drugs. 2019; (17): 570. doi: 10.3390/md17100570 31600939 PMC6835517

[15] Milanski M, Degasperi G, Coope A, Morari J, Denis R, Cintra DE, et al. Saturated fatty acids produce an inflammatory response predominantly through the activation of TLR4 signaling in hypothalamus: implications for the pathogenesis of obesity. J Neurosci. 2019; 29(2):359–70. 10.1523/JNEUROSCI.2760-08.2009. PMC6664935 19144836

[16] Senft D, Ronai ZA. UPR, autophagy, and mitochondria crosstalk underlies the ER stress response. Trends Biochem Sci. 2015; 40(3): 141–8. doi: 10.1016/j.tibs.2015.01.002 25656104 PMC4340752

[17] Liang W, Qi W, Geng Y, Wang L, Zhao J, Zhu K, et al. Necroptosis activates UPR sensors without disrupting their binding with GRP78. Proc Natl Acad Sci. 2021; 118(39): e2110476118. doi: 10.1073/pnas.2110476118 34544877 PMC8488584

[18] Yoshida H, Matsui T, Yamamoto A, Okada T, Mori K. XBP1 mRNA is induced by ATF6 and spliced by IRE1 in response to ER stress to produce a highly active transcription factor. Cell. 2001; 107(7): 881–9. doi: 10.1016/s0092-8674(01)00611-0 11779464

[19] Linton MF, Babaev VR, Huang J, Linton EF, Tao H, Yancey PG. Macrophage apoptosis and efferocytosis in the pathogenesis of atherosclerosis. Circ J. 2006; 80(11): 2259–2268. 10.1253/circj.CJ-16-0924. PMC5459487 27725526

[20] Leitman J, Shenkman M, Gofman Y, Shtern NO, Ben-Tal N, Hendershot LM, et al. Herp coordinates compartmentalization and recruitment of HRD1 and misfolded proteins for ERAD. Mol Biol Cell. 2014; 25(7): 1050–60. doi: 10.1091/mbc.E13-06-0350 24478453 PMC3967970

[21] Sano R, Reed JC. ER stress-induced cell death mechanisms. Biochim Biophys Acta. 2013; 1833(12): 3460–3470. doi: 10.1016/j.bbamcr.2013.06.028 23850759 PMC3834229

[22] Slodzinski H, Moran LB, Michael GJ, Wang B, Novoselov S, Cheetham ME, et al. Homocysteine-induced endoplasmic reticulum protein (herp) is up-regulated in parkinsonian substantia nigra and present in the core of lewy bodies. Clin Neuropathol. 2009; 28(5):333–43. . 19788048

[23] Ma Y, Hendershot LM. Herp is dually regulated by both the endoplasmic reticulum stress-specific branch of the unfolded protein response and a branch that is shared with other cellular stress pathways. J Biol Chem. 2014; 279(14):13792–9. 10.1074/jbc.M313724200. 14742429

[24] Finkelstein JD, Martin JJ. Homocysteine. Int J Biochem Cell Biol. 2000; 32(4):385–9. doi: 10.1016/s1357-2725(99)00138-7 10762063

[25] Sivanesan S, Taylor A, Zhang J, Bakovic M. Betaine and choline improve lipid homeostasis in obesity by participation in mitochondrial oxidative demethylation. Front Nutr. 2019; 10;5:61. 10.3389/fnut.2018.00061. PMC6048254 30042948

[26] Chang TY, Wu CH, Chang CY, Lee FJ, Wang BW, Doong JY, et al. Optimal dietary intake composition of choline and betaine is associated with minimized visceral obesity-related hepatic steatosis in a case-control study. Nutrients. 2022; 8;14(2):261. doi: 10.3390/nu14020261 35057441 PMC8779168

[27] Nathanielsz PW, Yan J, Green R, Nijland M, Miller JW, Wu G, et al. Maternal obesity disrupts the methionine cycle in baboon pregnancy. Physiol Rep. 2015; 3(11): e12564. doi: 10.14814/phy2.12564 26537341 PMC4673623

[28] Alves A, Bassot A, Bulteau AL, Pirola L, Morio B. Glycine metabolism and its alterations in obesity and metabolic diseases. Nutrients. 2019; 11(6): 1356. doi: 10.3390/nu11061356 31208147 PMC6627940

[29] Lima A, Ferin R, Bourbon M, Baptista J, Pavão ML. Hypercysteinemia, a potential risk factor for central obesity and related disorders in azores, portugal. J Nutr Metab. 2019; 20: 1826780. doi: 10.1155/2019/1826780 31321096 PMC6609363

[30] Narin F, Atabek ME, Karakukcu M, Narin N, Kurtoglu S, Gumus H, et al. The association of plasma homocysteine levels with serum leptin and apolipoprotein B levels in childhood obesity. Ann Saudi Med. 2005; 25(3):209–14. doi: 10.5144/0256-4947.2005.209 16119521 PMC6147986

[31] Wang WM, Jin HZ. Homocysteine: A potential common route for cardiovascular risk and dna methylation in psoriasis. Chin Med J (Engl). 2017; 130(16): 1980–1986. doi: 10.4103/0366-6999.211895 28776552 PMC5555134

[32] Jin Y, Amaral A, McCann A, Brennan L. Homocysteine levels impact directly on epigenetic reprogramming in astrocytes. Neurochem Int. 2016; 58(7): 833–8. 10.1016/j.neuint.2011.03.012. 21419186

[33] Hosoi T, Matsunami N, Nagahama T, Okuma Y, Ozawa K, Takizawa T, et al. 2-Aminopurine inhibits leptin receptor signal transduction. Eur J Pharmacol. 2006; 553(1–3):61–6. doi: 10.1016/j.ejphar.2006.09.044 17070518

[34] Couturier C, Jockers R. Activation of the leptin receptor by a ligand-induced conformational change of constitutive receptor dimers. J Biol Chem. 2003; 278(29): 26604–11. doi: 10.1074/jbc.M302002200 12734179

[35] Balland E, Dam J, Langlet F, Caron E, Steculorum S, Messina A, et al. Hypothalamic tanycytes are an ERK-gated conduit for leptin into the brain. Cell Metab. 2014; 19(2): 293–301. doi: 10.1016/j.cmet.2013.12.015 24506870 PMC3936883

[36] Duquenne M, Folgueira C, Bourouh C, Millet M, Silva A, Clasadonte J, et al. Leptin brain entry via a tanycytic LepR-EGFR shuttle controls lipid metabolism and pancreas function. Nat Metab. 2021; 3(8): 1071–1090. doi: 10.1038/s42255-021-00432-5 34341568 PMC7611554

[37] Banks WA, Farrell CL. Impaired transport of leptin across the blood-brain barrier in obesity is acquired and reversible. Am J Physiol Endocrinol Metab. 2003; 285(1): E10–5. doi: 10.1152/ajpendo.00468.2002 12618361

[38] Liu H, Du T, Li C, Yang G. STAT3 phosphorylation in central leptin resistance. Nutr Metab. 2021; 18(1): 39. doi: 10.1186/s12986-021-00569-w 33849593 PMC8045279

[39] Roujeau C, Jockers R, Dam J. Endospanin 1 determines the balance of leptin-regulated hypothalamic functions. Neuroendocrinology. 2019; 108(2): 132–141. doi: 10.1159/000494557 30326479

[40] Vauthier V, Swartz TD, Chen P, Roujeau C, Pagnon M, Mallet J, et al. Endospanin 1 silencing in the hypothalamic arcuate nucleus contributes to sustained weight loss of high fat diet obese mice. Gene Ther. 2014; 21(7): 638–44. doi: 10.1038/gt.2014.36 24784449

[41] Guo DF, Cui H, Zhang Q, Morgan DA, Thedens DR, Nishimura D, et al. The BBSome Controls Energy Homeostasis by Mediating the Transport of the Leptin Receptor to the Plasma Membrane. PLoS Genet. 2016; 29;12(2): e1005890. doi: 10.1371/journal.pgen.1005890 26926121 PMC4771807

[42] Wijesuriya TM, De Ceuninck L, Masschaele D, Sanderson MR, Carias KV, Tavernier J, et al. The Prader-Willi syndrome proteins MAGEL2 and necdin regulate leptin receptor cell surface abundance through ubiquitination pathways. Hum Mol Genet. 2017; 26(21): 4215–4230. doi: 10.1093/hmg/ddx311 28973533 PMC5886282

[43] Mazor R, Friedmann-Morvinski D, Alsaigh T, Kleifeld O, Kistler EB, Rousso-Noori L, et al. Cleavage of the leptin receptor by matrix metalloproteinase-2 promotes leptin resistance and obesity in mice. Sci Transl Med. 2018; 10(455): eaah6324. doi: 10.1126/scitranslmed.aah6324 30135249 PMC9678493

[44] Münzberg H, Flier JS, Bjørbaek C. Region-specific leptin resistance within the hypothalamus of diet-induced obese mice. Endocrinology. 2004; 145(11): 4880–9. doi: 10.1210/en.2004-0726 15271881

[45] Gao Q, Wolfgang MJ, Neschen S, Morino K, Horvath TL, Shulman GI, et al. Disruption of neural signal transducer and activator of transcription 3 causes obesity, diabetes, infertility, and thermal dysregulation. Proc Natl Acad Sci. 2004; 30;101(13): 4661–6. doi: 10.1073/pnas.0303992101 15070774 PMC384803

[46] Myers MG Jr, Leibel RL, Seeley RJ, Schwartz MW. Obesity and leptin resistance: distinguishing cause from effect. Trends Endocrinol Metab. 2010; 21(11): 643–51. doi: 10.1016/j.tem.2010.08.002 20846876 PMC2967652

[47] Škovierová H, Vidomanová E, Mahmood S, Sopková J, Drgová A, Červeňová T, et al. The molecular and cellular effect of homocysteine metabolism imbalance on human health. Int J Mol Sci. 2016; 17(10):1733. doi: 10.3390/ijms17101733 27775595 PMC5085763

[48] Ji C, Kaplowitz N. Hyperhomocysteinemia, endoplasmic reticulum stress, and alcoholic liver injury. World J Gastroenterol. 2004; 10(12): 1699–708. doi: 10.3748/wjg.v10.i12.1699 15188490 PMC4572253

[49] Zhang X, Qu YY, Liu L, Qiao YN, Geng HR, Lin Y, et al. Homocysteine inhibits pro-insulin receptor cleavage and causes insulin resistance via protein cysteine-homocysteinylation. Cell Rep. 2021; 37(2):109821. doi: 10.1016/j.celrep.2021.109821 34644569

[50] Deniz YILDIZ, Zuleyha CIVI, Yeliz CAKIR. Homocysteine Influx and Efflux: Participation of Erythrocytes in Homocysteine Homeostasis of the Plasma. GU J Sci. 2010; 23(3):249–253.

[51] Sato K, Nishii T, Sato A, Tatsunami R. Autophagy activation is required for homocysteine-induced apoptosis in bovine aorta endothelial cells. Heliyon. 2020;6(1):e03315. doi: 10.1016/j.heliyon.2020.e03315 32021943 PMC6994847

[52] Lipton SA, Kim WK, Choi YB, Kumar S, D’Emilia DM, Rayudu PV, et al. Neurotoxicity associated with dual actions of homocysteine at the N-methyl-D-aspartate receptor. Proc Natl Acad Sci U S A. 1997;94(11):5923–8. doi: 10.1073/pnas.94.11.5923 9159176 PMC20882

[53] Althausen S, Paschen W. Homocysteine-induced changes in mRNA levels of genes coding for cytoplasmic- and endoplasmic reticulum-resident stress proteins in neuronal cell cultures. Brain Res Mol Brain Res. 2000;84(1–2):32–40. doi: 10.1016/s0169-328x(00)00208-4 11113529

[54] Karatela RA, Sainani GS. Plasma homocysteine in obese, overweight and normal weight hypertensives and normotensives. Indian Heart J. 2009; 61(2):156–9. 20039500

[55] Vayá A, Rivera L, Hernández-Mijares A, de la Fuente M, Solá E, Romagnoli M, et al. Homocysteine levels in morbidly obese patients: its association with waist circumference and insulin resistance. Clin Hemorheol Microcirc. 2012; 52(1): 49–56. doi: 10.3233/CH-2012-1544 22460264

[56] Yun KU, Ryu CS, Oh JM, Kim CH, Lee KS, Lee CH, et al. Plasma homocysteine level and hepatic sulfur amino acid metabolism in mice fed a high-fat diet. Eur J Nutr. 2013; 52(1): 127–34. doi: 10.1007/s00394-011-0294-0 22209966

[57] Al-Bayyari N, Hamadneh J, Hailat R, Hamadneh S. Total homocysteine is positively correlated with body mass index, waist-to-hip ratio, and fat mass among overweight reproductive women: A cross-sectional study. Nutr Res. 2017; 48: 9–15. doi: 10.1016/j.nutres.2017.10.008 29246285

[58] Sinha JK, Ghosh S, Swain U, Giridharan NV, Raghunath M. Increased macromolecular damage due to oxidative stress in the neocortex and hippocampus of WNIN/Ob, a novel rat model of premature aging. Neuroscience. 2014;269:256–64. doi: 10.1016/j.neuroscience.2014.03.040 24709042

[59] Ghosh S, Sinha JK, Raghunath M. ’Obesageing’: Linking obesity & ageing. Indian J Med Res. 2019;149(5):610–615. 10.4103/ijmr.IJMR_2120_18. 31417028 PMC6702696

[60] Song M, Wang C, Yang H, Chen Y, Feng X, Li B, et al. P-STAT3 Inhibition Activates Endoplasmic Reticulum Stress-Induced Splenocyte Apoptosis in Chronic Stress. Front Physiol. 2020;11:680. doi: 10.3389/fphys.2020.00680 32714202 PMC7340136

[61] Gan L, Liu Z, Wu T, Feng F, Sun C. αMSH promotes preadipocyte proliferation by alleviating ER stress-induced leptin resistance and by activating Notch1 signal in mice. Biochim Biophys Acta Mol Basis Dis. 2017;1863(1):231–238. 10.1016/j.bbadis.2016.10.001. 27717825

[62] Kimura K, Yamada T, Matsumoto M, Kido Y, Hosooka T, Asahara S, et al. Endoplasmic reticulum stress inhibits STAT3-dependent suppression of hepatic gluconeogenesis via dephosphorylation and deacetylation. Diabetes. 2012;61(1):61–73. doi: 10.2337/db10-1684 22124464 PMC3237645

[63] Hansen IS, Schoonejans JM, Sritharan L, van Burgsteden JA, Ambarus CA, Baeten DLP, et al. ER stress abrogates the immunosuppressive effect of IL-10 on human macrophages through inhibition of STAT3 activation. Inflamm Res. 2019;68(9):775–785. doi: 10.1007/s00011-019-01261-9 31227842 PMC6667425

[64] Oslowski CM, Urano F. Measuring ER stress and the unfolded protein response using mammalian tissue culture system. Methods Enzymol. 2011;490:71–92. doi: 10.1016/B978-0-12-385114-7.00004-0 21266244 PMC3701721

[65] Wang L, Ryoo HD, Qi Y, Jasper H. PERK Limits Drosophila Lifespan by Promoting Intestinal Stem Cell Proliferation in Response to ER Stress. PLoS Genet. 2015;11(5):e1005220. doi: 10.1371/journal.pgen.1005220 25945494 PMC4422665

[66] Meares GP, Liu Y, Rajbhandari R, Qin H, Nozell SE, Mobley JA, et al. PERK-dependent activation of JAK1 and STAT3 contributes to endoplasmic reticulum stress-induced inflammation. Mol Cell Biol. 2014;34(20):3911–25. doi: 10.1128/MCB.00980-14 25113558 PMC4187715

[67] Bao Y, Liang W, Ye Y, Yi B. PERK-Dependent Activation of the JAK2/STAT3 Pathway Contributes to High Glucose-Induced Extracellular Matrix Deposition in Renal Tubular Epithelial Cells. Int J Endocrinol. 2021;2021:8475868. doi: 10.1155/2021/8475868 34335747 PMC8315854

[68] Liang Y, Liang L, Liu Z, Wang Y, Dong X, Qu L, et al. Inhibition of IRE1/JNK pathway in HK-2 cells subjected to hypoxia-reoxygenation attenuates mesangial cells-derived extracellular matrix production. J Cell Mol Med. 2020;(22):13408–13420. doi: 10.1111/jcmm.15964 33043579 PMC7701502

[69] Gonnella R, Gilardini Montani MS, Guttieri L, Romeo MA, Santarelli R, Cirone M. IRE1 Alpha/XBP1 Axis Sustains Primary Effusion Lymphoma Cell Survival by Promoting Cytokine Release and STAT3 Activation. Biomedicines. 2021;9(2):118. doi: 10.3390/biomedicines9020118 33513694 PMC7912693

[70] Argemí J, Kress TR, Chang HCY, Ferrero R, Bértolo C, Moreno H, et al. X-box Binding Protein 1 Regulates Unfolded Protein, Acute-Phase, and DNA Damage Responses During Regeneration of Mouse Liver. Gastroenterology. 2017;152(5):1203-1216.e15. 10.1053/j.gastro.2016.12.040. 28082079

