# Supplementary figures and images for: Homocysteine causes neuronal leptin resistance and endoplasmic reticulum stress
Source: PLoS One. 2022 Dec 13;17(12):e0278965. doi: 10.1371/journal.pone.0278965 (PMC9746958; doi:10.1371/journal.pone.0278965)

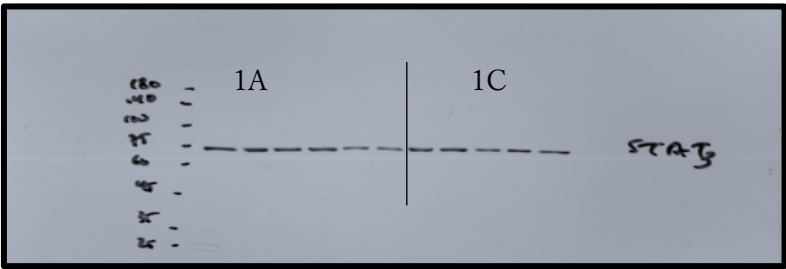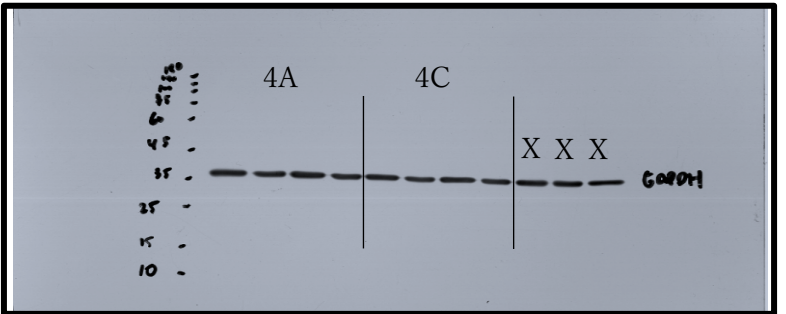

Fig. 5A

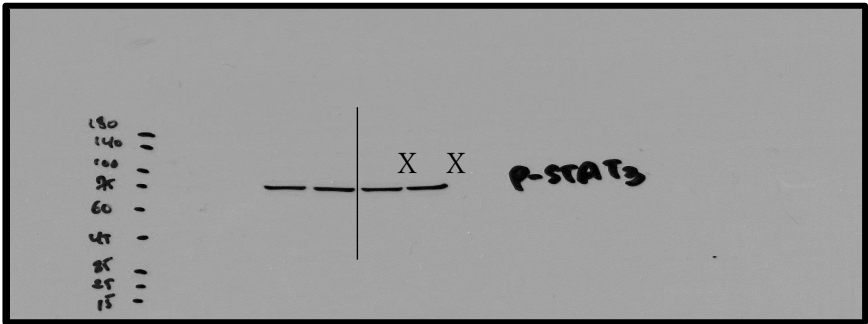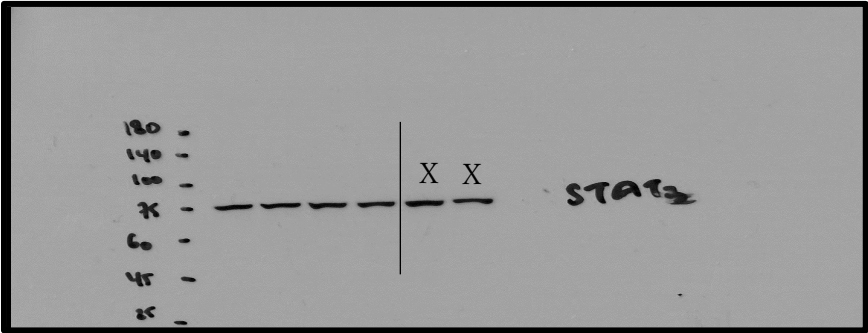

Fig.5C

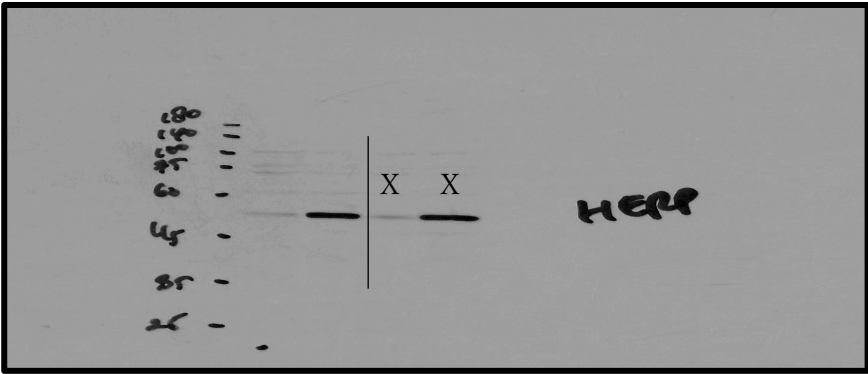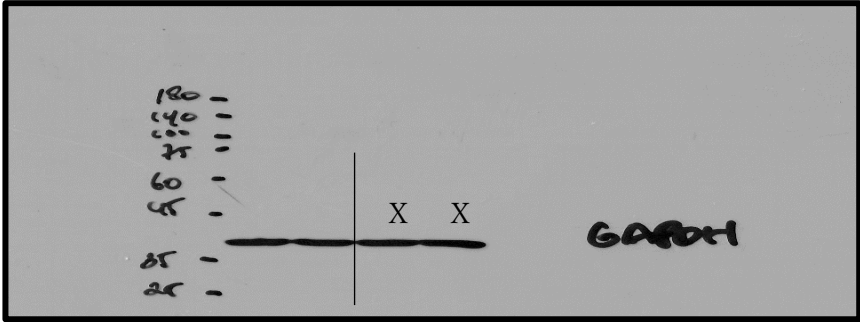

Supplement: S1 Raw images — (PDF) [file pone.0278965.s001.pdf]
